# Preventable Pediatric Stroke via Vaccination?

**DOI:** 10.15844/pedneurbriefs-29-10-3

**Published:** 2015-10

**Authors:** Craig A. Press, Mark S. Wainwright

**Affiliations:** 1 Ruth D. & Ken M. Davee Pediatric Neurocritical Care Program, Ann & Robert H. Lurie Children's Hospital of Chicago, Chicago, IL; 2 Departments of Pediatrics and Neurology, Northwestern University Feinberg School of Medicine, Chicago, IL

**Keywords:** Pediatric Stoke, Infection, Vaccination

## Abstract

Investigators from the Vascular Effects of Infection in Pediatric Stroke (VIPS) group studied the risk of arterial ischemic stroke (AIS) associated with minor infection and routine childhood vaccinations.

Investigators from the Vascular Effects of Infection in Pediatric Stroke (VIPS) group studied the risk of arterial ischemic stroke (AIS) associated with minor infection and routine childhood vaccinations. Children from 1 month to 18 years of age presenting with AIS (n=355) were compared to age-matched controls presenting for a routine health visit or due to trauma (n=354). Etiology of the strokes comprised arteriopathy (46%), cardioembolic stroke (21%), a prothrombotic condition (9%), another risk factor (5%) or were idiopathic (5%). Patients with AIS were more likely to have reported infection in the week prior with an odds ratio (OR) of 6.3 (95% [CI] 3.3–12). This effect diminished by one month. The most common infections and symptoms were upper respiratory (URI) (50%) with cough and fever. Preceding infection was common across all subtypes of AIS. Patients with a recent infection were more likely to be younger with a median age of 4.0 compared to 9.1. Notably, children who were reported to have some, few, or no vaccinations had an increased rate of AIS 7.9% vs 1.2% (OR of 7.3 (95% [CI] 2.5–21)). Both the effect of recent infection and vaccination remained significant in a multivariable logistic regression model adjusting for age, sex and season of enrollment. [1]

COMMENTARY. This paper builds on previous retrospective cohort studies supporting a relationship between infection and AIS [2, 3] in particular for arteriopathy [4]. There are many proposed mechanisms by which recent infection could increase the risk of AIS: by inflammation causing a prothrombotic state, direct arterial wall infection and inflammation, or dehydration. However, the finding that AIS was less likely for patients who received a vaccination the week prior suggests that systemic inflammation is not the entire story. Further, URIs were the most common infection as opposed to gastroenteritis (making dehydration as a trigger less compelling), and suggesting local head and neck infection as the more specific risk factor.

The juxtaposition of recent infection increasing the risk of AIS while more comprehensive vaccination reducing this risk, is striking. If the vaccinations were against known causes of common minor infections a causative association would be more plausible. Several possibilities could explain this discrepancy: 1) vaccine-preventable diseases are responsible for the increased AIS risk, 2) vaccination changes the immune milieu altering the inflammatory response which leads to AIS or 3) vaccinations indirectly prevent additional infections (i.e. URI) thus decreasing AIS risk. Further research using Next Generation Sequencing, proteomic and RNA expression analysis may identify interactions between the immune system and the infectious microbiome increasing childhood AIS risk. The association of infection with increased risk for all AIS types may indicate a common mechanism or is a marker of an underlying vulnerability to infection and AIS.

While further study is needed, these results suggest that obtaining a detailed infectious and vaccination history for children with AIS is important, and may help identify the causes of otherwise unexplained AIS. This unclear causal relationship between infection and stroke will require further study as is already proposed by the VIPS investigators [5] and we look forward to these results.
